# Application of radiomics feature captured from MRI for prediction of recurrence for glioma patients

**DOI:** 10.7150/jca.65366

**Published:** 2022-01-04

**Authors:** Canyu Liu, Yujiao Li, Xiang Xia, Jiazhou Wang, Chaosu Hu

**Affiliations:** 1 Department of Radiation Oncology, Suzhou Dushu Lake Hospital, Dushu Lake Hospital Affiliated to Soochow University, Medical Center of Soochow University, Suzhou, 225200, China; 2 Department of Radiation Oncology, Fudan University Shanghai Cancer Center; Department of Oncology, Shanghai Medical College, Fudan University, Shanghai, 200032, China

**Keywords:** gliomas, MRI-based radiomics, recurrence

## Abstract

**Purpose:** This study aimed to develop and validate a recurrence prediction of glioma patients through a radiomics feature training and validation model.

**Patients and methods:** In this study, the prediction model was developed in a training cohort that consisted of 88 patients from January 2014 to July 2017 with pathologically confirmed gliomas. Their pre-radiotherapy and recurrence brain magnetic resonance imaging (MRI) images were collected, and the radiomics features were extracted. Clinical factors including age, gender, WHO grade, Isocitrate dehydrogenases (IDH) mutation status and treatment after surgery were collected. The least absolute shrinkage and selection operator (LASSO) regression model was conducted for data dimension reduction, feature selection, and radiomics feature analysis. Internal validation was assessed. An independent validation cohort contained 41 consecutive patients from August 2017 to December 2018. Furthermore, multivariable logistic regression analysis was used to develop the predicting model by combining the radiomics signature and independent clinical factors.

**Results:** In total, 129 patients were included, among which 40 patients had recurrence. The median follow-up time was 27.4 (range, 2.6-79.2) months. We compared the tumor regions radiomics difference between the recurrence and non-recurrence patients. The radiomics signature was associated with the event of recurrence (P < 0.001 for both training and validation cohorts, respectively). The training model showed good discrimination with a C-index of 0.7578 (95%CI: 0.6549-9.8608) through internal validation on T1 contrast-enhanced magnetic resonance imaging, and a consistent trend in calibration. In the validation cohort, the model also showed good discrimination (C-index, 0.6925, 95%CI: 0.5145-0.8705) and good calibration. In the other two sequences of MRI (T1WI, T2WI), the validation model also showed positive results. Meanwhile, radiomics feature and clinical factors were significantly prognostic for recurrence (P value <0.05, respectively).

**Conclusion:** We identified the radiomics feature derived from brain MRI that presented potential in predicting recurrence in glioma patients. This could be beneficial to risk stratification for patients. Further investigation is necessary to include expanded sample size investigation and external multicenter validation.

## Introduction

Glioma is the most prevalent primary brain cancer in human beings, of which about 80% are malignant gliomas 1, 2. Lower grade gliomas including World Health Organization (WHO) grade I and II are less common and predominantly affects younger adults 3. On the contrary, high-grade glioma (WHO grade III and IV), especially glioblastoma multiforme (GBM), is the most common and aggressive subtype of gliomas with an annual incidence rate of 3.2 per 100000 population 4. The disease of malignant gliomas can directly affect patients' quality of life and cognitive function with a poor prognosis 5.

For WHO grade I tumors without adverse prognostic factors, we often take an observational approach after a completely resection surgery. On the other hand, those high-grade patients, accounting for the tumor invasiveness, a maximal safe resection followed by a combination of radiotherapy (Intensity-modulated radiation therapy (IMRT) in daily fraction of 2 Gy given 5 days per week for 6 weeks, for a total of 60 Gy) and/or chemotherapy (concurrent temozolamide (TMZ) followed by 6-12 cycles of TMZ) is the standard care treatment 6. Never the less, the median survival is only 14.6 months 6. Majority of patients recur after a short time and about 90% recurrence are within the radiation filed 7. The event of recurrence is the complex problem in the treatment process for gliomas. Therefore, early prediction of recurrence patients and recurrence locations can result in early aggressive treatments which can reduce the risk of recurrence and improve prognosis.

There are two aims in this study. First, we will investigate whether radiomics features could predict the recurrence patient by pre-operation MRI images. Second, we try to will identify the recurrence region from patient tumor.

### Patiens and Methods

**Figure 1:**
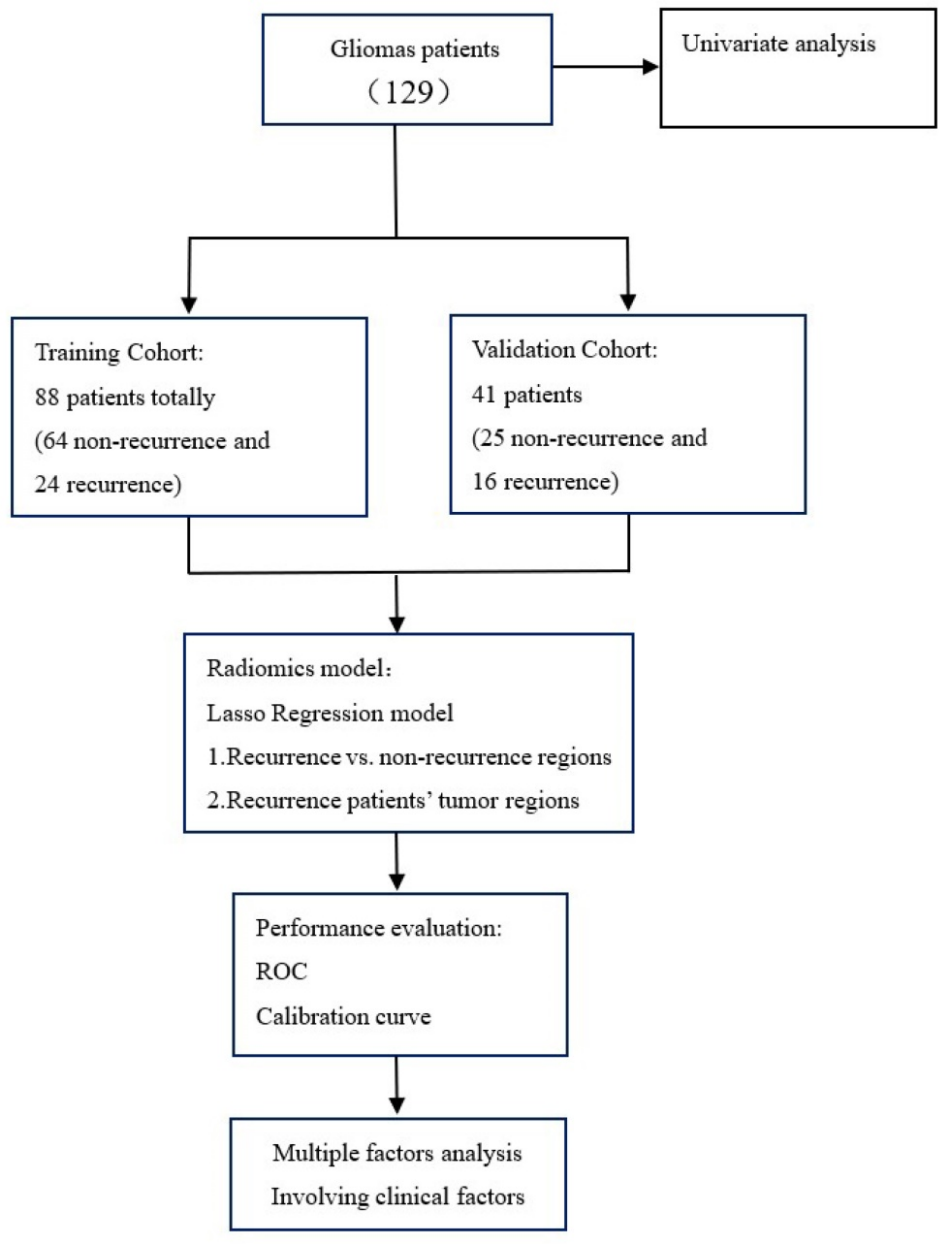
Study flowchart.

Our study was conducted according to the flow chart in Figure 1. All of the radiomics feature were extracted from MRI T1weighted image (T1WI), T2 weighted image (T2WI), T1 contrast-enhanced. Two radiomics model including patient model and region models was established by least absolute shrinkage and selection operator (LASSO). The model performance was evaluated by ROC and calibration curve. For the patient model, we also evaluate the relationship between the radiomics feature and clinical factors by using multi-factor analysis of variance.

### Patients

129 patients with glioma confirmed by pathology from January 2014 to December 2018 in Fudan University Shanghai Cancer Center (FUSCC) were enrolled retrospectively in this study. The inclusion criteria included: 1) had a histopathologic diagnosis of glioma according to 2016 WHO classification, 2) had preoperative and follow-up 1.5T MR imaging including T1WI, T2WI, contrast-enhanced T1WI, 3) underwent the standard treatment, concurrent radiochemotherapy and adjuvant TMZ after maximal surgical resection. The exclusion criteria included: 1) poor MR imaging quality; 2) follow-up loss. All patients underwent surgery and postoperative intensity modulated radiotherapy (IMRT). Overall survival (OS) was defined as time in months between first treatment and death. The retrospective study strictly obeyed the principles of the Declaration of Helsinki. This retrospective study was approved by the Fudan University Shanghai Cancer Center Institutional Review Board and all methods were performed in accordance with the guidelines and regulations of this ethics board. All participants signed their informed consent after being fully informed of the purpose and content of this study.

### Treatment protocol

For treatment planning, the MD Anderson Cancer Center target policy was used 8*.* For grade III-IV glioma, the total dose of radiotherapy given was 60 Gy; five fractions of 2Gy per week were given for 6 weeks. The tumor shown on the preoperative MRI scan, not including comprehensively T2-weighted sequence hyperintense signal, was irradiated with a margin of 2 cm, and the target volume was reduced to a margin of 1.5 cm around the tumor after 50 Gy. Temozolomide was given orally at 75 mg/m^2^ on days 1-42 of radiotherapy and at 150 mg/m² per day for 5 days, repeated every 28 days (one cycle). Patients with grade II glioma received standard radiotherapy treatment, which consisted of IMRT 54 Gy (30 × 1.8 Gy once daily, 5 days per week, 6 weeks).

### MR Image Acquisition, Region of Interest (ROI) Segmentation and Radiomic Feature Extraction Methodology

All MRI scans were performed on a 1.5T MR scanner (Magnetom Skyra, Siemens Healthcare, Erlangen, Germany) with a 16-channel phase-array body coil. The MR protocol included an oblique axial high resolution T1-weighted TSE sequence, an oblique axial high resolution contrast-enhanced T1-weighted TSE sequence, an oblique axial high resolution T2-weighted TSE sequence, a sagittal T2-weighted turbo spin echo (TSE) sequence, and an oblique axial diffusion-weighted imaging (DWI) sequence.

Recurrence/progressive tumor volume (RTV) was defined as post-treatment gadolinium enhancement on post-RT T1-weighted MRI and delineated by an experienced radiation oncologist (D.C.W.) on the MRI datasets. RTVs were delineated without the knowledge of the planning GTVs and BTVs to avoid bias.

The target delineation of radiotherapy was delineated by one radiation oncologist and reviewed by another senior radiation oncologist on the MIM system and both radiation oncologists had more than ten years of clinical experience in glioma. After contouring, the DICOM images and contours were exported to MatLab (MathWorks, Natick, MA) for feature extraction and analysis. The contoured regions of the images were cropped from the whole patient axial high resolution contrast-enhanced T1WI, axial high resolution T1WI, and high-resolution T2WI scans by creating a binary mask based on the contouring. After tumor segmentation, 257 radiomic features were extracted from each MRI images. These features can be divided into the following groups: 1) gray level co-occurrence matrix (GLCM), 2) gray level run-length matrix (GLRLM), 3) wavelet GLCM, 4) wavelet GLRLM, 5) histogram, 6) geometry, and 7) fractal. All radiomic features for each patient were extracted from their axial high resolution contrast-enhanced T1WI, axial high resolution T1WI, and high-resolution T2WI images with a calculation algorithm executed in MatLab R2015a.

### Model develop and validation

A univariate analysis was performed to analyze the impact of each radiomics features. For binary variables, a chi-square test was used. A Cox regression analysis was used to analyze the relation between the radiomics features and clinical factors.

Patients were divided into training cohort (January 2014 to July 2017) and validation cohort (August 2017 to December 2018) according to time for first visit. The prior portion comprised the training set (88), the latter portion composed the validation set (41). We use the patient model and region model for recurrence prediction. In the patient model, we use the whole tumor region for radiomics calculation. For the region model, we analysed the 40 recurrence patients' recurrence and non-recurrence regions. The least absolute shrinkage and selection operator (LASSO) method, which is a supervised machine-learning method 9, and a regression analysis method that performs both variable selection and regularization to enhance the prediction accuracy and interpretability of the statistical model it produces were used to select the most useful predictive features from the dataset, and the predictive model was established using the LASSO method. Therefore, the lambda selection of the LASSO model was based on the larger patient cohort, and the selection method was 10-fold cross validation. The final LASSO model was based on the whole training dataset. The area under the curve (AUC) of the receiver operating characteristic (ROC) curve of the prediction model, sensitivity, and specificity were calculated to evaluate the predictability of the model with the validation dataset. All the statistical analyses were conducted with R software (version 3.3.1; http://www.Rproject.org), and statistical significance levels are indicated by two-sided p values with α set at 0.05.

## Results

### Patient characteristics and univariate analysis

**Figure 2:**
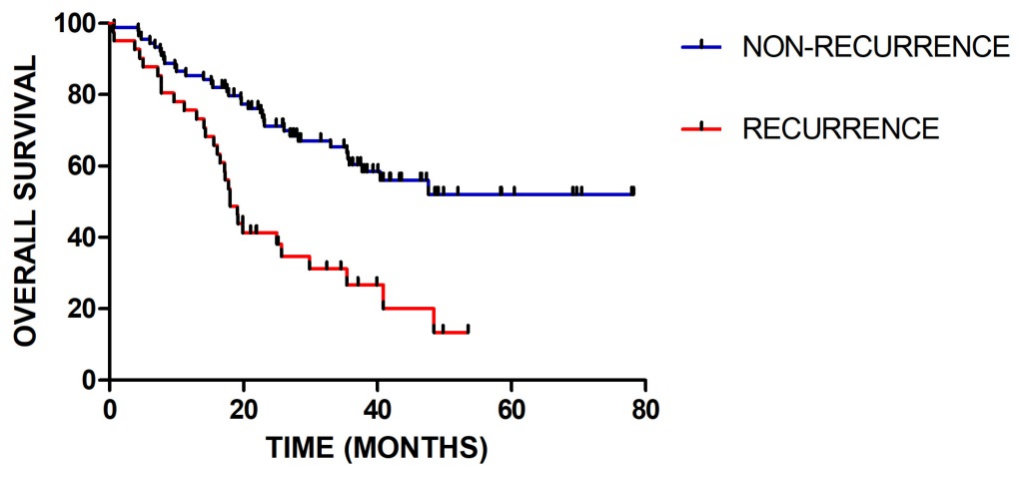
Kaplan-Meier survival curves for patients with recurrence or without recurrence (P = 0.001)

In total, 129 patients were included, among which 40 patients had recurred. The median follow-up time was 27.4 (range 2.6-79.2) months. For clinical factor, the gender and concurrent chemotherapy have no significant influence. There are significant differences in age (p=0.037), WHO grade (p=0.001), IDH status (p=0.03), radiotherapy-interruption (p=0.017) and adjuvant chemotherapy (p=0.009) between patients with recurrence and without recurrence. As shown in Figure 2, there is significant difference in the overall survival between patients with recurrence and without recurrence (p=0.001). Detailed patients' characteristics and baseline information in the training and validation cohorts are provided in Table 1.

### Prognostic value of radiomics signature

We implemented univariate analyses to assess the prognostic value of radiomics and clinical factors associated with recurrence during surveillance. There was a significant difference in local recurrence in the radiomics signature (p<0.001) and age (p=0.043). The radiomics-based prognostic models were superior in recurrence prediction to the clinical factors (P <0.05).

### Performance of patient model

**Figure 3:**
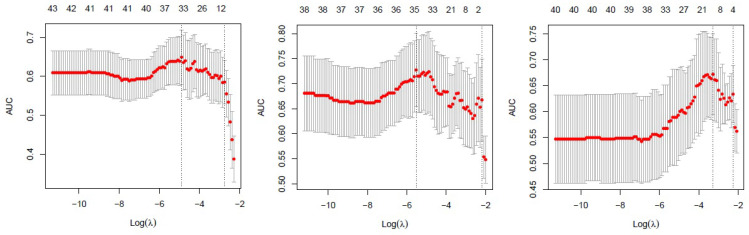
Feature selection using the least absolute shrinkage and selection operator (LASSO) binary logistic regression model. Tuning parameter (λ)selection in LASSO used 5-fold cross-validation via minimum criteria. The area under the receiver operating characteristic (AUC) was plotted versus log(λ) separately for T1WI(left), T2WI(median), contrast-enhanced T1WI(right) of patient model. T1WI, T1 weighted image; T2WI, T2 weighted image.

**Figure 4:**
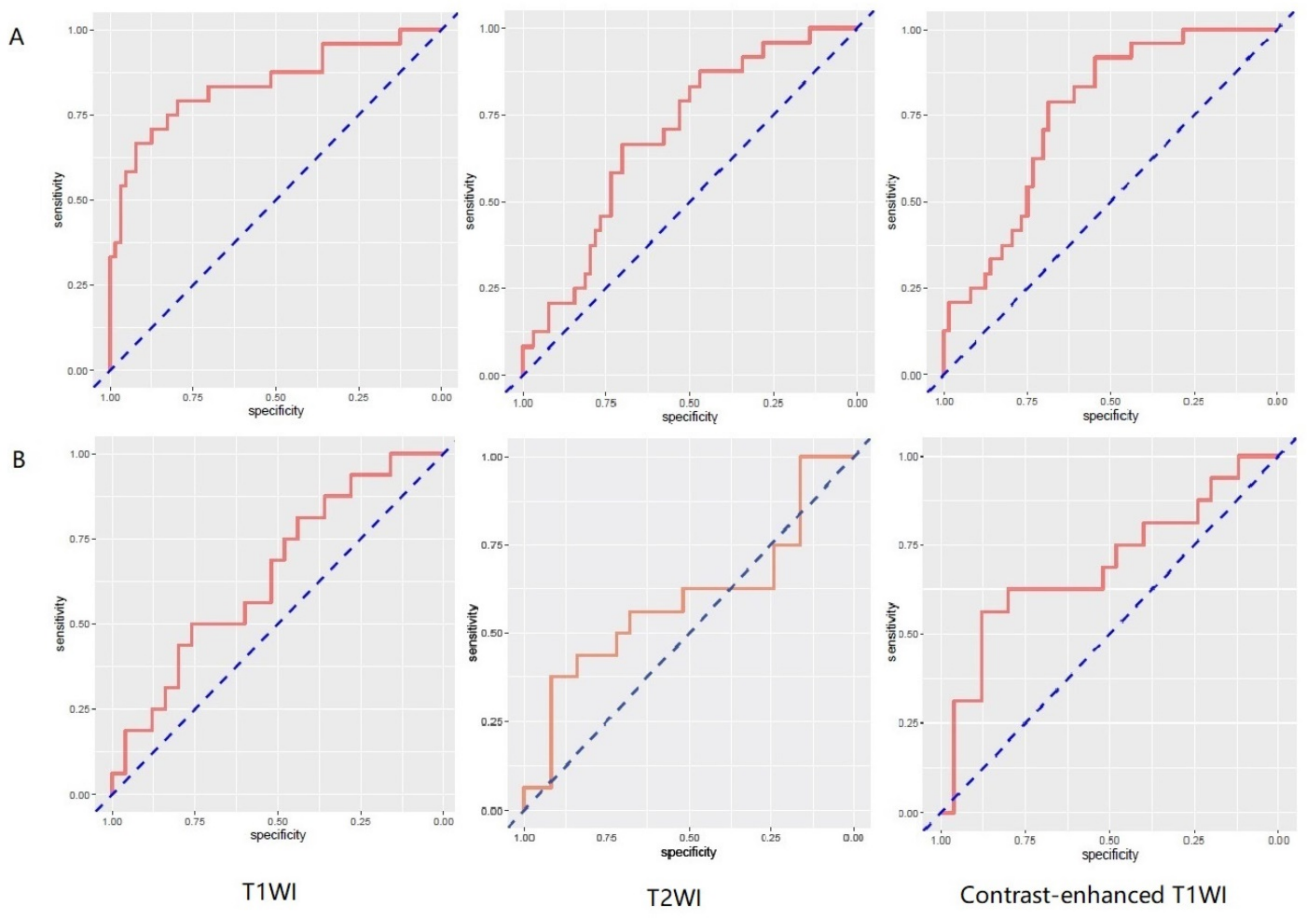
ROC curves of radiomics signature in predicting recurrence among glioma patients for patient model. A and B represent separately for training and validation cohorts. From left to right, the images represent T1WI, T2WI, contrast-enhanced T1WI in turn. AUC was defined as area under curve. For an effective regression model (AUC >0.5), the closer AUC is to 1.0, the better the model. ROC, receiver operating characteristic curve; AUC, area under curve. T1WI, T1 weighted image; T2WI, T2 weighted image.

257 radiomics feature of ROI (region of interest) for each radiomic signature were reduced to 44 potential predictors on the basis of 88 patients in the training cohort. The feature selection was demonstrated in Figure 3. The receiver operating characteristic curve for training and validation cohorts was presented in Figure 4. The AUC values for each radiomics signature of three regular MR image sequence (T1WI, T2WI, contrast-enhanced T1WI) were showed in Table 2.

**Figure 5:**
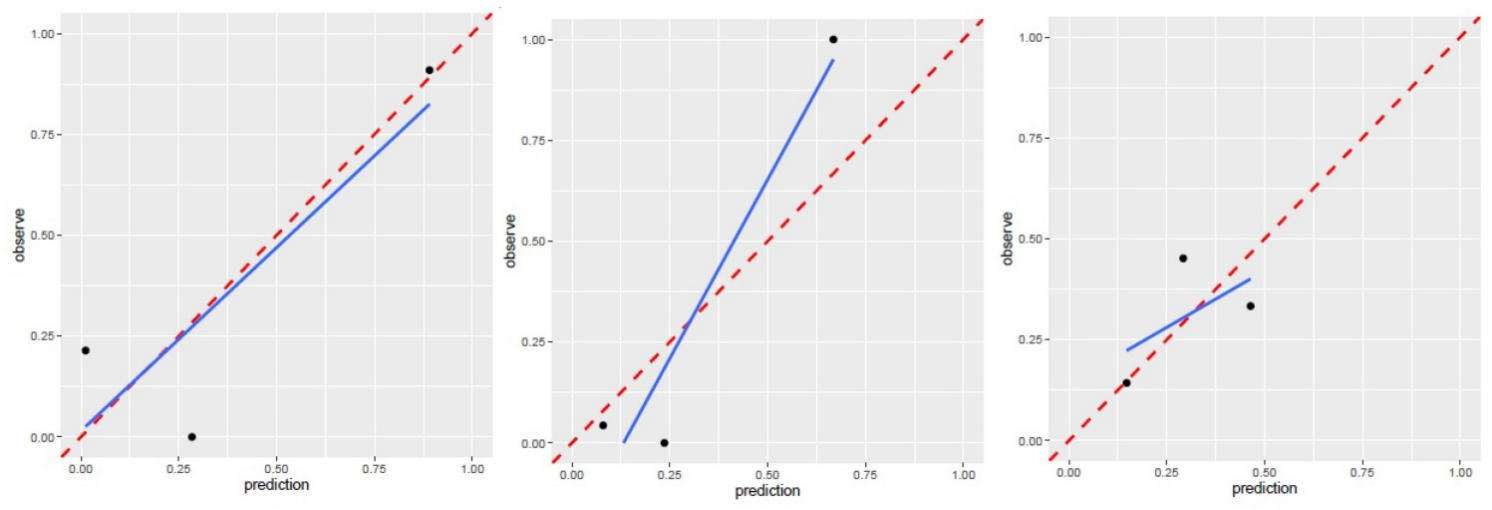
Calibration curve for T1WI(left), T2WI(median), contrast-enhanced T1WI(right) of patient model. T1WI, T1 weighted image; T2WI, T2 weighted image. Performance of region model

The AUC of patient model on validation dataset was 0.6475 (0.4744-0.8206) for T1WI, 0.5925 (0.3976-0.7874) for T2WI, 0.6925 (0.5145-0.8705) for contrast-enhanced T1WI radiomics feature. Good calibration was observed for the prediction probability for recurrence patients (Figure 5).

**Figure 6:**
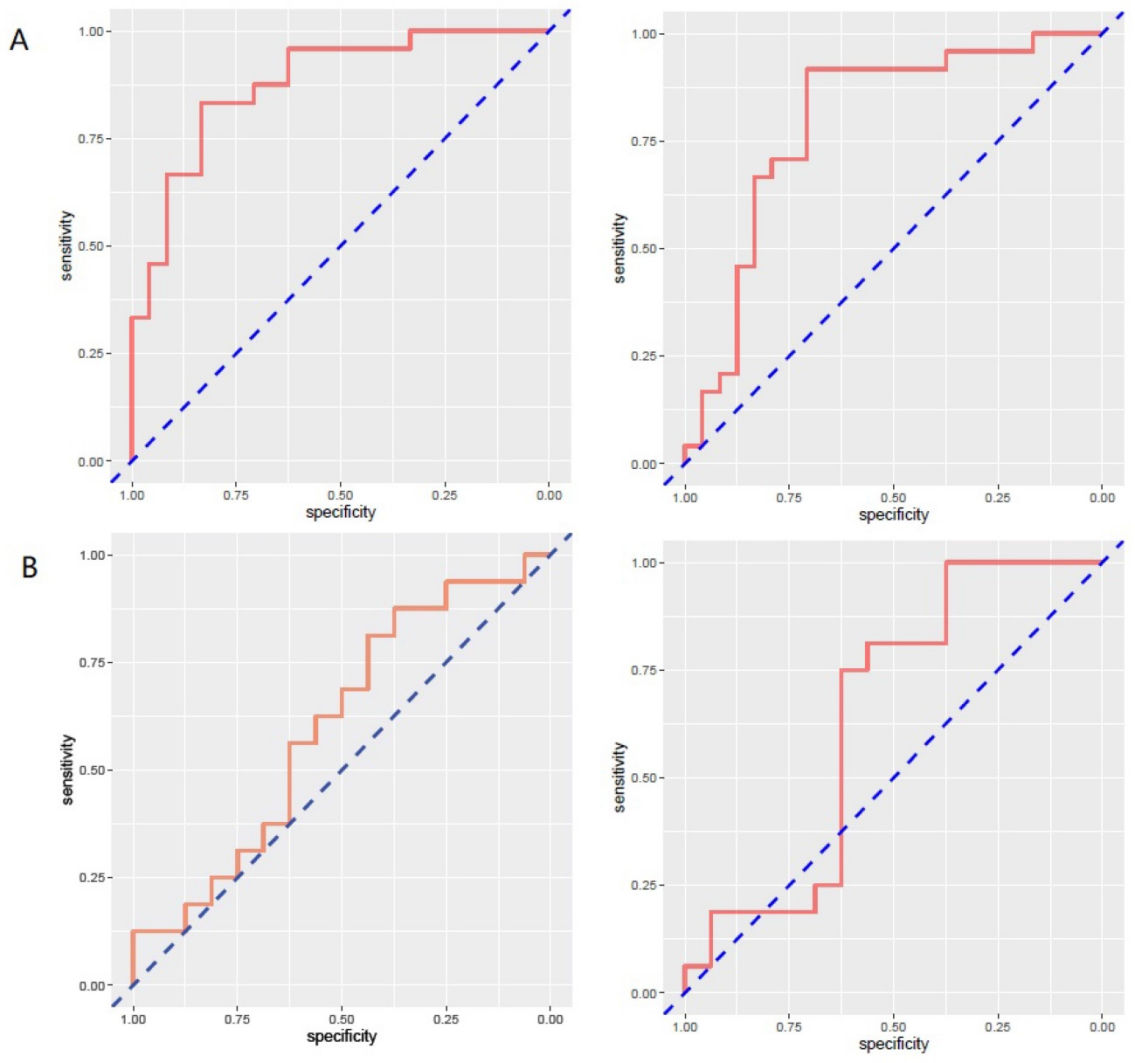
ROC curves of radiomics signature in predicting recurrence in region for region model. A and B represent separately for training and validation cohorts. From left to right, the images represent T1WI, T2WI in turn. AUC was defined as area under curve. For an effective regression model (AUC >0.5). ROC, receiver operating characteristic curve; AUC, area under curve; T1WI, T1 weighted image; T2WI, T2 weighted image.

The limitation of the sample size for recurrence patients lead to no positive results for contrast-enhanced T1WI radiomics feature. The receiver operating characteristic curve for training and validation cohorts was presented in Figure 6. The AUC values for each radiomics signature of three regular MR image sequence (T1WI, T2WI) were showed in Table 3.

**Figure 7:**
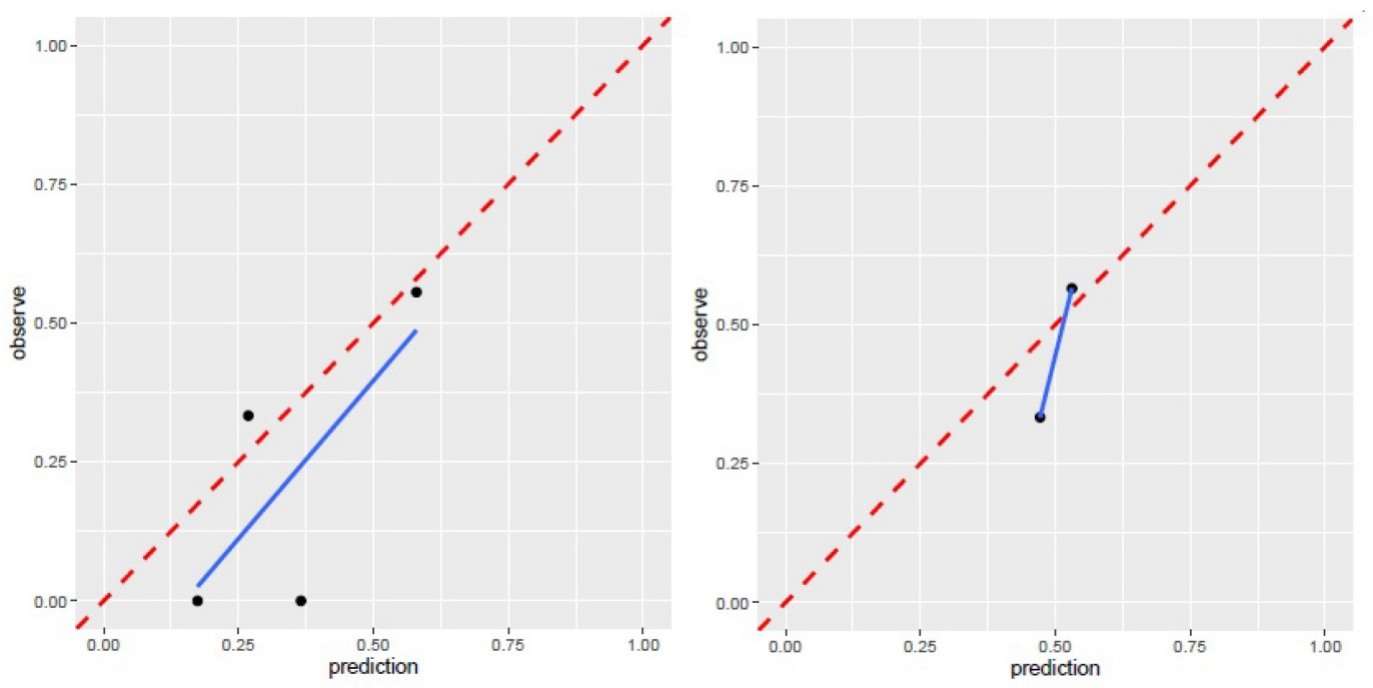
Calibration curve for T1WI, T2WI of region model. T1WI, T1 weighted image; T2WI, T2 weighted image.

The AUC of region model on validation dataset was of 0.6061 (95% CI: 0.3982-0.8049) for T1WI, 0.7951 (95% CI: 0.4323-0.8489) for T2WI (Fig.6). The calibration curve for the probability of local recurrence region prediction demonstrated good agreement between prediction and observation in the prediction model (Figure 7).

## Discussion

In this study, we develop and validated a radiomics model for the pretreatment individualized prediction of recurrence in patients with gliomas especially those with high-grade gliomas. The prediction model incorporated the item of the radiomics signature of MR image, since MRI is one of the important imaging techniques for diagnosis and follow-up in gliomas patients 10-12. A robust modeling method was implemented to get the final model. This study is the first attempt for creating a prediction model of recurrence in glioma patients which may guide the treatment strategy.

**Figure 8:**
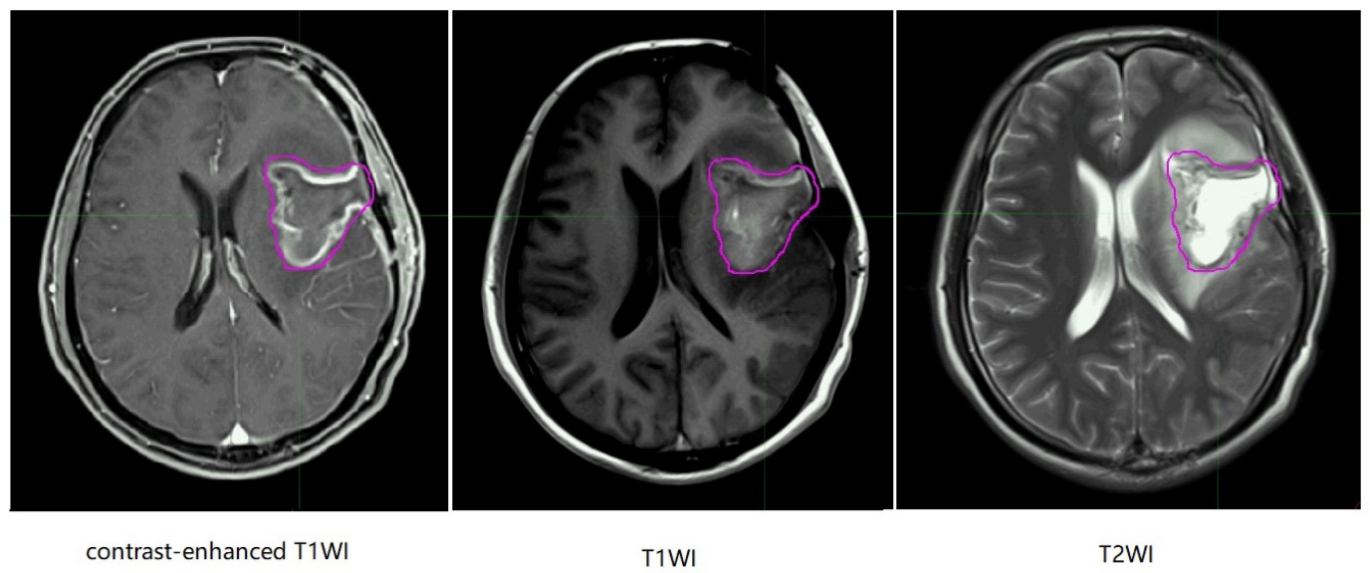
Representative non-recurrence patient's regular brain MRI images, the purple line represents gross tumor volume. T1WI, T1 weighted image; T2WI, T2 weighted image.

**Figure 9:**
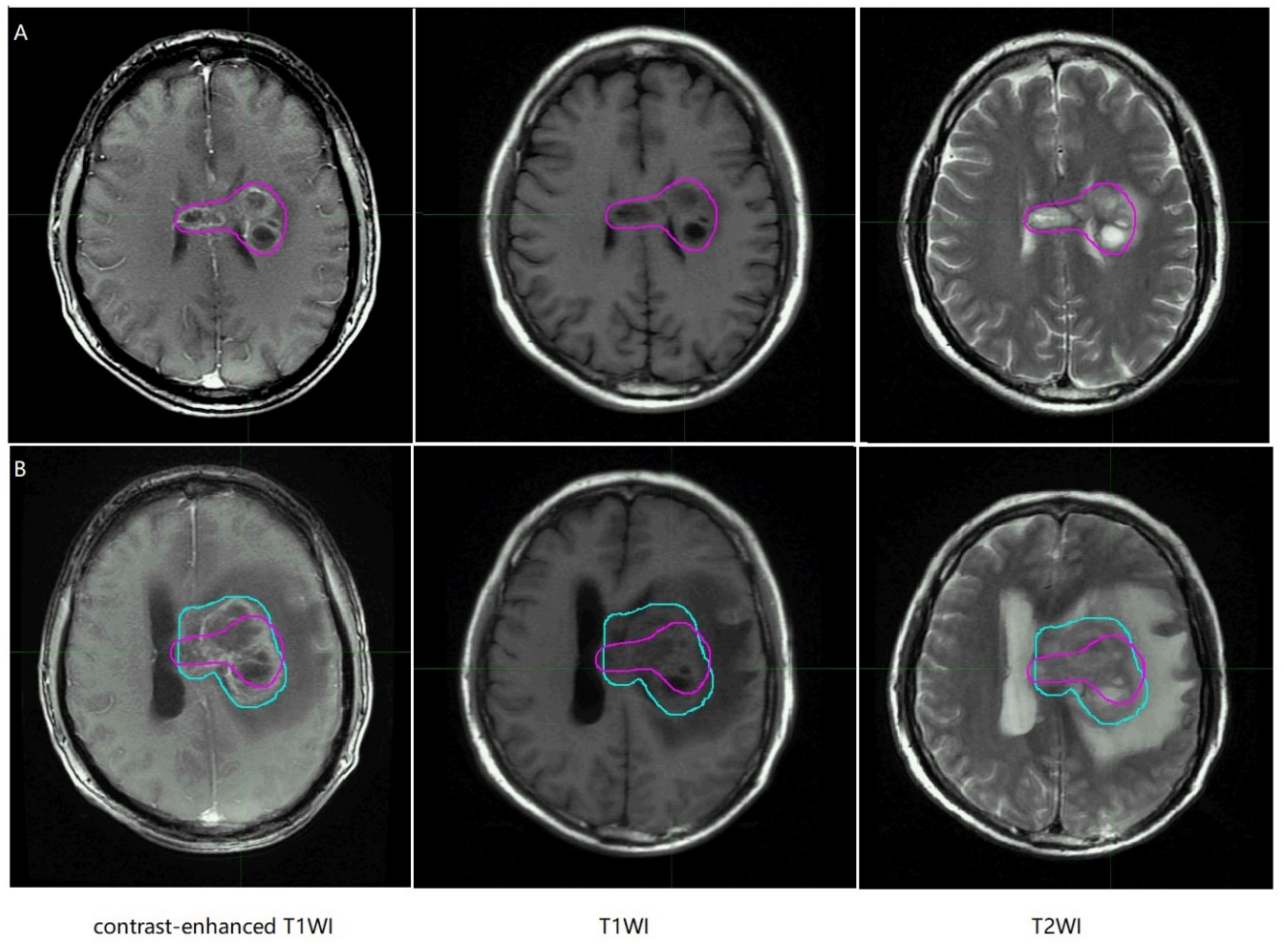
Representative recurrence patient's regular brain MRI images, the purple line and the blue line represent gross tumor volume of primary tumor and gross tumor volume of recurrent tumor. T1WI, T1 weighted image; T2WI, T2 weighted image.

In the MR images of glioma patients, we delineated the gross tumor volume and recurrence tumor volume according to the regular MR images. As showed in Figure 8 and Figure 9, we tried to find the radiomics signature of tumor region without the peritumoral edema region.

The radiomics feature shows discrimination among recurrence and non-recurrence patients. In variable selection, collinearity causes competition among predictors and make arbitrary decisions in choice. The radiomics signature demonstrated adequate discrimination in the training cohort, which was improved in the validation cohort.

The results show that the clinical factors such as age, IDH mutation status, and WHO grade were significantly prognostic for recurrence in glioma patients. IDH-1 mutation predicts a better prognosis. Although the standard treatment for malignant glioma patients are applied, most patients suffer from local recurrence, which was similar to previous studies 13.

The recurrence for glioma patients especially for GBM makes clinical control difficult. Previous studies reported that hypo-fractionation may have no significant differences in OS compared with standard IMRT in elderly patients 14. There was no clear evidence suggest that the hypo-fractionated IMRT can improve local tumor control and OS. Re-resection for recurrence has been shown to have an impact on survival 15. Intervention for recurrence glioblastoma patients: re-resection, systemic therapy, re-radiation may not benefit all the patients 16. Therefore, early prediction of recurrence is very important and treatment must be individualized.

Imaging such as MRI, CT and positron emission tomography (PET) can be used for tumor diagnosis, guiding therapy, and predicting clinical outcomes 12, 17, 18. A few studies in differentiating recurrence of gliomas had been reported. ^11^C-methionine (11C-MET) PET was reported that can be used for identifying recurrent brain tumor by radiomics approach 19. Kai et al. found the advantages of ^18^F-fluorodeoxyglucose (^18^F-FDG) positron emission tomography (PET) radiomics features in identifying recurrence by integrating ^18^F-FDG (PET), ^11^C-methionine (11C-MET) PET and magnetic resonance images 20. However, these studies mainly focused on the treatment assessment in recurrent malignant gliomas by radiomics approach and most of them didn't take clinical factors into account 21, 22. The purpose and significance of our study is early detection of relapse-prone patients and areas. In this study, we use radiomics features extracted out of MRI to study the recurrence characteristic. By creating a prediction model, the recurrence of malignant glioma patients can be identified through the validation model and early prediction of recurrence location can be achieved.

There are some limitations in this study. Considering the aggressiveness of gliomas and lack of quality -in-life for patients, most patients lose to follow-up after surgery. Thus, the sample size of this study is limited. An expanded sample size and multicenter validation are necessary for further investigation in order to verify the results of our study. Secondly, the genomic characteristics, such as 1p/19q, MGMT, EGFR, were not fully considered 23-25. It is necessary for further radiogenomic analysis. Finally, our study was implemented on limited regular MRI sequence (T1WI, T2WI, contrasted-enhanced T1WI), the predictive radiomics value of other functional MRI sequence such as T2 Flair, DWI could be further explored. Due to the limitation of pathology information, more than half of the patients had no MGMT gene promoter detection. Therefore, we excluded the "Methylation of the MGMT gene promoter" in the considered clinical factors.

## Conclusion

We identified one wavelet texture feature derived from brain MRI that presented potential in predicting recurrence in glioma patients. This preliminary finding allows possibility in exploring risk prediction models for early identification of recurrence for such patients. Further investigation is necessary to include expanded sample size investigation and external multicenter validation.

## Figures and Tables

**Table 1:** Patient characteristics and baseline information

Characteristic	Training Cohort		P value	Validation Cohort	
	Recurrence	Non-recurrence		Recurrence	Non-recurrence
Gender			0.501		
Male	17(70.8)	34(53.1%)		8(50%)	16(64%)
Female	7(29.2)	30(46.9%)		8(50%)	9(36%)
Age			0.037		
<=54	7(29.2%)	36(56.3%)		8(50%)	15(60.0%)
>54	17(70.8%)	28(43.7%)		8(50%)	10(40.0%)
WHO Grade			0.001		
I	0(0%)	0(0%)		0(0%)	5(20%)
II	1(4.2%)	17(26.6%)		0(0%)	7(28%)
III	6(25.0%)	22(34.4%)		3(18.8%)	13(52%)
IV	17(70.8%)	25(34.1%)		13(81.3%)	0(0%)
IDH status			0.03		
Wide type	10(41.7%)	15(23.4%)		14(87.5%)	10(40.0%)
Mutation type	5(20.8%)	16(25.0%)		2(12.5%)	12(48.0%)
Unknown	9(37.5)	33(51.6%)		0(0%)	3(12.0%)
Radiotherapy-interruption			0.017		
Yes	3(12.5%)	1(1.6%)		2(12.5%)	1(4.0%)
No	21(87.5%)	63(98.4%)		14(87.5%)	24(96.0%)
Concurrent chemotherapy			0.092		
Yes	22(91.7%)	48(75.0%)		1(6.2%)	24(96.0%)
No	2(8.3%)	16(25.0%)		15(93.8%)	1(4.0%)
Adjuvant chemotherapy			0.009		
Yes	19(79.2%)	32(50.0%)		13(81.2%)	18(72.0%)
No	5(20.8%)	32(50.0%)		3(13.8%)	7(28.0%)

Abbreviations: P value was derived from the univariate logistic regression analyses between each of the variables and recurrence events.

**Table 2:** Prediction power analysis in patient model

	Training cohort	Validation cohort
T1WI	0.8424 (0.7371-0.9478)	0.6475 (0.4744-0.8206)
T2WI	0.6914 (0.5739-0.8089)	0.5925 (0.3976-0.7874)
Contrast-enhanced T1WI	0.7578 (0.6549-0.8705)	0.6925 (0.5145-0.8705)

**Table 3:** Prediction power analysis in region model

	Training cohort	Validation cohort
T1WI	0.8785 (0.7819-0.975)	0.6016 (0.3982-0.8049)
T2WI	0.5 (0.659-0.9319)	0.7951 (0.4323-0.8489)
Contrast-enhanced T1WI	NA	NA
